# Implementing an Oral Health Intervention for People Experiencing Homelessness in Scotland: A Participant Observation Study

**DOI:** 10.3390/dj6040068

**Published:** 2018-12-01

**Authors:** Laura Beaton, Isobel Anderson, Gerry Humphris, Andrea Rodriguez, Ruth Freeman

**Affiliations:** 1 Dental Health Services Research Unit, School of Dentistry, University of Dundee, Dundee DD1 4HN, Scotland, UK; a.rodriguez@dundee.ac.uk (A.R.); R.E.Freeman@dundee.ac.uk (R.F.); 2 Housing Studies, Faculty of Social Sciences, University of Stirling, Stirling FK9 4LA, Scotland, UK; isobel.anderson@stir.ac.uk; 3 School of Medicine, Medical & Biological Sciences, University of St Andrews, North Haugh, St Andrews KY16 9TF, Scotland, UK; gmh4@st-andrews.ac.uk

**Keywords:** homeless persons, oral health, delivery of health care, dental health services

## Abstract

Smile4life is an intervention aimed at improving the oral health of people experiencing homelessness in Scotland. The purpose of this research was to determine how this intervention was being translated from guidance into action. Data concerning Smile4life working practices were collected in three NHS Boards using participant observation. Fieldnotes taken during these observations were analysed using content analysis. This analysis revealed that there were working alliances between the oral health practitioner, the Third Sector staff, and the homeless service users, and that these alliances were affected by various barriers and enablers. The observation sessions also highlighted variations in working practices.

## 1. Introduction

Homelessness is a multi-dimensional experience characterised not merely by the lack of a roof over one’s head but also by physiological and emotional deprivation [1]. Being homeless can be “impoverishing and isolating” and is often associated with physical and mental ill-health [2]. Indeed, as Scottish Government statistics show, 42% of those who were assessed as homeless during 2015–2016 were found to have one or more additional support needs, including mental ill-health, drug or alcohol dependency as well as medical condition(s) [3]. This suggests that this group of people had experienced multiple exclusion homelessness, which can be defined as the experience of homelessness in addition to one or more of the following: institutional care (e.g., prison, hospital or being a looked after child); street activities such as begging; or substance misuse [4].

In addition to the physical health of people experiencing homelessness, there is evidence that this population have poor oral health. Previous studies have found that homeless populations have a high prevalence of bleeding gums, calculus and periodontal inflammation [5,6,7]. Research has also found that people experiencing homelessness often have a poor record of dental attendance and unmet treatment needs [6]. In addition, this population has been found to suffer from dental anxiety and poor oral health related quality of life [5]. 

People experiencing homelessness face many health inequalities—for example, they have a higher risk of death than the general population, as well as higher rates of suicide and depression [8]. Poor oral health could also be seen as a contributing factor to the health inequalities faced by the homeless population: the Groundswell Healthy Mouths report found that “participants were regularly facing issues with their oral health that were making it difficult for them to live fulfilled lives” [9] (p. 2). In addition, those that had oral health problems reported being “handicapped” with 21% of participants reporting they felt “completely unable to function” because of their oral health, compared to 1% of the general population [9] (p. 2). Many reported turning to alcohol and drugs to help them cope with their dental pain, which, in turn, exacerbates other health needs [8].

The Scottish Government recognised the health needs of the Scottish homeless population in 2005, with the Health and Homelessness Standards, designed to improve the health of people experiencing homelessness and to tackle health inequalities [10]. This was followed in the same year by the Dental Action Plan, which recognised people experiencing homelessness as a priority group that required tailored oral healthcare [11]. Both documents called for the National Health Service (NHS) to take steps to address the general and oral health of people experiencing homelessness in Scotland. In Scotland, the NHS is comprised of 14 Boards, each linked to a geographical area of the country, and provides primary and secondary healthcare to the whole population, regardless of their ability to pay, and is free at the point of delivery [12]. This need for tailored health messages was strengthened by evidence from a systematic review and meta-analysis that concluded that messages that were tailored to the health needs of patients were effective in promoting behaviour change [13]. In 2012, the Scottish Government renewed its commitment to the oral health of people experiencing homelessness in the Priority Groups Strategy, which emphasised the need for accessible dental services and preventive oral health programmes [14].

In response to the Scottish Government’s policies, an oral health intervention, called Smile4life, was developed to address the oral health needs of the homeless population in Scotland. It was developed from the evidence-base gleaned from a national survey of 853 participants that was conducted during 2008–2009. This sample population was shown to have had poorer oral and psychosocial health when compared to the general population of Scotland [15]. Qualitative interviews also took place with 34 people experiencing homelessness, highlighting that maintaining good oral health was not always practically possible when homeless. Furthermore, oral health was often not a priority, as evidenced by poor dental attendance [15]. Together, the quantitative and qualitative findings from the Smile4life report led to the development of the Smile4life intervention and accompanying Guide for Trainers [16], a resource intended to be used by National Health Service (NHS) and Third Sector staff to assist in the training of their staff to deliver evidence-based oral health messages to their service users. For the purposes of this research, the Third Sector refers to voluntary or charity organisations or community groups working with homeless service users, providing support and/or accommodation.

The Guide for Trainers provided oral health practitioners and Third Sector staff with: an overview of homelessness and oral health, including barriers and enablers to oral health care; information about oral health (e.g., specific problems, access to care, and preventive care advice); information about the common risk factor approach to oral health; and information about the Smile4life intervention itself, including guidelines for specific roles that practitioners could adopt, motivational interviewing techniques, how to deliver tailored oral health advice, and how to support behaviour change. The Guide for Trainers, and the Smile4life intervention itself, was launched in 2012 [16]. 

A theory-based process evaluation of the implementation of the Smile4life intervention began in 2013. Regular telephone interviews took place with NHS staff responsible for the implementation. The evaluation found that it took NHS Boards an average of 16 months to implement the Smile4life intervention following the launch of the Guide for Trainers training package. The results of this evaluation highlighted various barriers to successful implementation, particularly a lack of resources (staffing, time constraints), and poor engagement between the NHS and the Third Sector [17]. Factors that facilitated implementation included motivated practitioners and a willingness to engage with other organisations [17].

While this process evaluation illustrated factors that act as barriers and/or facilitators to successful implementation, much remained unknown about how and why NHS and Third Sector organisations implement Smile4life. Therefore, this study aimed to answer the question: how is Smile4life being translated from national guidance into practice? 

To begin to answer this question, it was crucial to ascertain how the Smile4life intervention was currently being delivered within NHS Boards. In order to do so, it was necessary to observe the behaviours associated with the delivery of Smile4life.

## 2. Materials and Methods

### 2.1. Sample and Recruitment

Sampling was purposive, with all three NHS Boards being selected because they had taken part in the earlier previous evaluation of Smile4life and had demonstrated contrasting levels of experience and a variety of approaches in delivering oral/dental health services to people affected by homelessness, allowing for a theoretical sampling [17,18,19]. Furthermore, the NHS Boards also varied in the number of people assessed as being homeless, with Board 3 having 7685, Board 2 having 6056 and Board 1 having 2379 in the most recent statistics from the Scottish Government [20].

The recruited individuals were all NHS employees who worked directly with service users, offered training to Third Sector staff, and delivered oral health education. Their job titles varied depending on the NHS Board of employment and included oral health educators and dental health support workers. Oral health educators promote and raise awareness of oral health issues amongst the homeless population and any Third Sector staff that work in the homelessness sector. They deliver oral health advice, provide support and maintain links with homeless organisations. The role of a dental health support worker is community-based, supporting homeless individuals directly by providing oral health advice, signposting to relevant services, making dental appointments and accompanying patients to the dentist, if required. For the purposes of this article, these participants are referred to as “oral health practitioners”. 

### 2.2. Ethical Considerations 

Ethical approval was applied for and granted by the University Research Ethics Council at the University of Dundee (UREC 15098). Consent forms had to be read and signed before any observation could take place and all data were anonymised.

### 2.3. Data Collection

Participant observation was chosen as the data collection method for this research, in order to reveal the existing relationships between the oral health practitioners and Third Sector practitioners and the homeless service users they interact with, as well as variations in the working practices of the oral health practitioners. Participant observation would allow the researcher to observe first hand these interactions and variations as they happened, rather than relying on second-hand accounts from the oral health practitioners themselves about their working practices. Furthermore, using participant observation for this research was in line with Taylor-Powell and Steele’s guidelines on when participant observation is appropriate, e.g., “when you are trying to understand ongoing behaviour, process, unfolding situation or event” or “when written or other data collection procedures seem inappropriate” [21] (p. 1). In addition, participant observation is recognised as being a “valuable approach for community health research”, providing an opportunity for researchers to become more involved in the community of the person(s) being observed, whereby “the informants are more likely to disclose their real beliefs and perspectives” [22] (p. 4). 

Detailed field notes were taken to record what was heard and what was seen. The field notes recorded the date, time and location, as well as any other relevant contextual information, and were written in a narrative style. Notes were made of everything that seemed relevant as Taylor-Powell and Steele noted “in some situations, observing what does not happen may be as important as observing what happens” [21] (p. 3). Each additional observation session was then informed by the ones that preceded it, allowing for theoretical sampling.

As Jorgensen stated, “it is important at the outset of inquiry to remain open to the unexpected” [23] (p. 82). Therefore, the observation was based on a combination of structured and unstructured formats—there were pre-identified items that should be looked for, as well as space to report anything relevant but unexpected [21]. This allowed an insight into the existing relationship between the oral health practitioners, Third Sector practitioners and homeless service users, and reveal variations in the working practices of the oral health practitioners. It also revealed whether practitioners were following the national guidance in Smile4life, concerning oral health and homelessness. 

### 2.4. Data Analysis

Content analysis was chosen as the method by which to analyse the data collected from the observation sessions. This is a form of data analysis that is understood to be a “systematic and objective means of describing and quantifying phenomena” [24] (p. 108). It “involves establishing categories, (and) systematic linkages between them” [25] (p. 467). 

Before any data were collected, the researcher reflected on the aim of the observation sessions—to explore how Smile4life was being delivered within the NHS Boards—and had identified several key topics that would be specifically looked for. The researcher also considered the recommendations made by Mays and Pope that observers should aim “to record exactly what happened, including his or her own feelings and responses to the situations witnessed” [18] (p. 184). The key issues the researcher aimed to observe primarily concerned dialogues about oral health, and the role and skills of the practitioner. The researcher also noted the physical context, any general observations that did not fit into any of these questions, and then her own reflections of the session that had been observed. 

These questions helped to select specific areas to examine and analyse, as part of the preparation stage of content analysis, e.g., how practitioners engaged with service users and Third Sector staff, and how they have chosen to implement Smile4life. The data were then open coded—reading through all the field notes and identifying recurrent categories, based on existing knowledge and reflecting on the research question. Memos, written in the margins, were used to note emerging ideas and reflections concerning the data [26]. The process was then repeated, to test and refine and revise categories, with similar sub-categories being grouped together where appropriate [18,27]. 

## 3. Results

Data were collected over a ten-month period, beginning in November 2015, and ending in August 2016. Three observation sessions were conducted in Board 1, two in Board 3 and four in Board 2. In Boards 2 and 3, these were whole day sessions, where the researcher accompanied the oral health practitioners to a series of different locations as they went about a normal Smile4life working day. In Board 1, this approach was not possible, so sessions lasted approximately 1.5–2 h—the time that a mobile dental unit spent at a location treating patients. Data were collected at the three locations over a series of sessions until saturation had been reached, i.e., when the researcher had witnessed the full range of services offered by the oral health practitioner being observed. More information about each location visited is provided in Table 1. More details about the practitioners are included in Table 2. 

From the initial coding, one overarching theme emerged: a working alliance between the oral health practitioner and (i) the Third Sector staff, and (ii) the service users. Evidence of this alliance is presented below, followed by an exploration of the barriers and enablers to a positive working alliance.

The concept of a working alliance originated in psychoanalysis, where it is understood to be part of a therapeutic relationship between a health professional and patient. More generally, it is the relationship between a person who wants to make a change, and another person who can help them to make that change [28]. Bordin stated that this working relationship was “key to the change process” [28] (p. 252), and compared the relationship to that of a parent and child or teacher and pupil. Bordin explained that the working alliance is comprised of three factors: “an agreement on goals, an assignment of task or series of tasks, and the development of bonds” [28] (p. 253). In relation to Smile4life, these three factors could be interpreted as: improving the oral health of homeless service users; promoting Smile4life and encouraging service users to attend oral health sessions; and a strong relationship between the oral health practitioner and the Third Sector staff. 

It became clear that, with regard to the delivery of Smile4life, the working alliance went beyond the traditional dyadic relationship of a health professional and a patient—it also involved a third element: the Third Sector staff. Triadic relationships first came to prominence in the work of the sociologist Georg Simmel. While Simmel had written about this in 1908, Hill and McGrath [29] argued that it did not gain wider attention until 1950 when his work was definitively translated by Wolf. Simmel stated that when three elements are present “each one operates as an intermediary between the other two” [30] (p. 135). The third person can have three potential roles: (1) a mediator who “deprives conflicting claims of their affective qualities because it neutrally formulates and presents these claims to the two parties involved”; (2) a non-partisan: one party who facilitates the “concord of two colliding parties” or an arbiter who “balances… contradictory claims against one another”; or (3) a “tertius gaudens”, a person that can benefit from the conflict of the other two parties within a triad [30] (pp. 146–154). Simmel also highlighted that, in some situations, a third person joining an existing dyad could be seen as an intruder [30]. Indeed, when two parties are present, there can be no majority but, when a third party joins, the group dynamic can shift to two against one, “revealing emergent power relations” [29] (p. 53).

**Figure 1:**
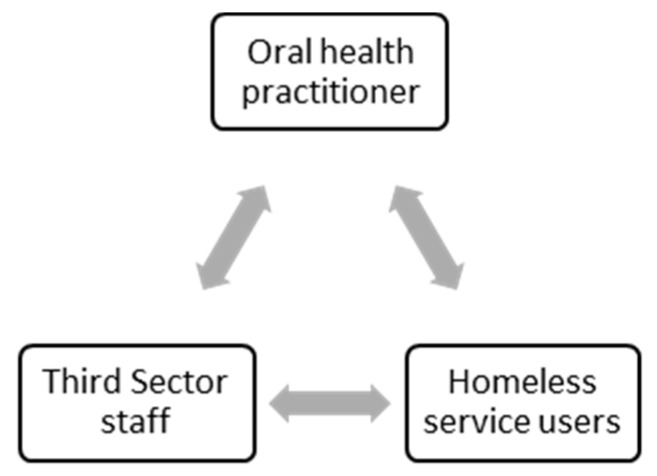
The Smile4life triadic working alliance.

Within the Smile4life triadic working alliance, there are three principle relationships: (1) the oral health practitioner and the service user; (2) the oral health practitioner and the Third Sector member of staff; and (3) the Third Sector member of staff and the service user. These are discussed in turn below, and are illustrated in Figure 1.

### 3.1. Alliance 1: Oral Health Practitioner and Service User

For the oral health practitioners that were observed, interacting directly with homeless service users was their main role with regards to delivering the Smile4life intervention (as opposed to training Third Sector staff). Therefore, this alliance needed to be strong, in order for the oral health practitioner to engage with the service users and address their oral health needs, and for the service user to be interested and receptive to the oral health information being discussed. However, this alliance was affected by numerous barriers, which are discussed below.

#### 3.1.1. Barriers

In many of the locations where the oral health practitioners were observed, it was often the oral health practitioner that had the initiative to approach the service user about their oral health, and this was often opportunistic, with the oral health practitioners seizing upon any opportunity to engage with service users.

“Practitioner 4 would go up to tables with men having breakfast and say who she was and why she was there, but just to make them aware—it was up to the individuals to approach her if they wanted to”

*(Observation 8)*

However, as this excerpt from the field notes highlights, the oral health practitioners are dependent on the service users taking an interest in what they have to say. Often, this is not the case—there was occasionally disinterest or hostility from the service users, who were usually not interested in receiving oral health advice. In Boards 2 and 3, many of the interactions with service users are opportunistic, with the oral health practitioner approaching the service user to talk about oral health, rather than the other way around. In one instance, a service user at a drop-in did not want the toothbrush pack from the practitioner because the toothpaste contained fluoride which he considered to be a “neurotoxin”. The practitioner tactfully explained the benefits of fluoride, but did not stress the point because the service user was getting angry and argumentative with her.

An additional barrier that prevented the formation of strong working alliance between the oral health practitioners and the service users was the space the oral health practitioner was given by the service in which to deliver Smile4life. As Table 1 shows, in many locations, the oral health practitioner was told to set up in a large communal area, often a canteen—a space in which there is the potential to reach many service users. However, in seven of the services visited, the oral health practitioner was put in a space that meant the service users must approach them, i.e., a medical room, or a meeting room away from the communal spaces. This blocked engagement attempts of any kind, by preventing the oral health practitioner from approaching the service users directly. Furthermore, the rooms the oral health practitioners were put in were predominantly chosen by the staff, and were not always appropriate for the delivery of Smile4life.

*“We were put in the medical room along the corridor from the office, but there was no opportunity for Practitioner 4 to approach any of the residents. We only saw service users if they specifically wanted to talk about their oral health or if they had walked past the room and wanted to see who we were.”*

*(Observation 9)*

In the case of Board 1, the space that the oral health practitioners were in was also not ideal, but this was not due to the staff within the Third Sector services they were attending. All data collection took place within the Mobile Dental Unit, which was a small confined space. Outside of the dental surgery section of the Unit there was a waiting area with enough space for approximately two people—any more than two and it began to feel crowded. This meant that it was not the most appropriate place for impromptu oral health advice or longer discussions about oral health with service users. 

The observations at the Mobile Dental Unit (MDU) highlighted an additional barrier to the working alliance between the oral health practitioners and the service users—that there are risks involved in delivering Smile4life and that the service users can be unpredictable and occasionally violent. On a number of occasions, disruptive service users were observed barging into the Unit, demanding to be seen by a dentist. In these instances, the oral health practitioner was the one who acted as a gatekeeper, preventing the service users from accessing the dental surgery section of the Unit.

“When I was there today two service users opened the MDU door from the outside and barged in”

*(Observation 3)*

“When I arrived there were two women outside the front door (of the MDU) shouting.”

*(Observation 4)*

Perceptions of there being risks associated with Smile4life delivery were strengthened by the repeated use of safety measures, such as alarms or radios, in the services visited while observing the oral health practitioners. In most instances, these devices were given to the oral health practitioner by the Third Sector staff before they proceeded to interact with service users—the researcher never observed an emergency alarm or radio being used. Indeed, it became apparent that the oral health practitioners did not feel they were at risk, despite Practitioner 1 having a good reason to be concerned about her safety. She told the researcher that on a previous visit to a drop-in she had been bitten on the hand by a service user, while she had been discussing oral health with him. This event was then reported to Datix, the NHS’s incident reporting tool. However, when she saw that he was present at the session we were attending, she did not avoid him and spoke with him again. At the same session, Practitioner 1 had warned the researcher beforehand to be careful about her handbag, because some of the service users had been known to steal.

#### 3.1.2. Enablers

It became clear that there were two key factors in overcoming these barriers and enabling a successful working alliance to be formed between the oral health practitioners and the service users: the skills and attitudes of the oral health practitioners and the use of incentives. 

With regard to the oral health practitioners themselves, the researcher observed that they needed to be confident, and in some respects, fearless. Indeed, the field notes frequently reflect this:

*“Working in the mobile dental unit would not be for everyone—you need to be confident and thick-skinned”*

*(Observation 3)*

*“Practitioner 1 is confident and appears quite fearless, putting up with language/behaviour that would not be tolerated in a normal clinic.”*

*(Observation 6)*

*“I get the impression that Practitioner 4 and the other practitioners I have observed… do not see the risk involved in their job, or they just see it as part of the job… it is also possible that after a while doing this kind of work they stop seeing it as risky”*

*(Observation 9)*

It became apparent that working on Smile4life was not something that would suit everyone—the oral health practitioners themselves said as much.

*“The dental team spoke about how they felt that working in the MDU would not suit everyone”*

*(Observation 3)*

*“I believe that not everyone is suited to doing Practitioner 1’s job—personality aside, you need to be fast-thinking, tough-skinned, a bit fearless, and approachable.”*

*(Observation 6)*

In addition to confidence, the oral health practitioners also had to be flexible in their interactions with service users, in particular, tolerating disruptive behaviour, for a working alliance to be sustained. In Board 1, the oral health practitioner, and the dentist and dental nurse who worked in the Mobile Dental Unit, described some service users as being disruptive, trying to flirt with them and one man who had taken off his t-shirt to show them his tattoos. Other service users swore or used offensive language. The oral health practitioners admitted that they tolerated this kind of behaviour from the patients they saw in the MDU, but would not normally do so, with patients they saw in their usual clinics. In the case where the patient was flirting and removing his clothes, they did not seem fazed by this. It became apparent over the course of the observation sessions that the success of engagement attempts was closely associated with the individual oral health practitioner, and their personality, experience, communication skills and how they perceived their job role and responsibilities.

From this, it appears that, for the oral health practitioner to be successful in implementing Smile4life, they needed to be flexible, not just in their working hours (e.g., working in the evenings) but also in tolerating disruptive behaviours from patients, while remaining non-judgmental and not taking undue risks. They must tailor their approach to the needs of the individual service user.

*“Practitioner 1 is very experienced and upfront with all service users—not visibly fazed by service users’ admissions or behaviours”*

*(Observation 1)*

*“The MDU dentist admitted that she is aware that she acts differently with patients in the MDU than she would with regular patients—she is not as formal, more likely to speak to them in the same way they speak to her… she is quite matter-of-fact”*

*(Observation 3)*

Furthermore, the researcher observed that the oral health practitioners have to be very sensitive and empathetic, to strengthen the working alliance—often service users will share information about their lives, and their past experiences, and practitioners must listen and respond appropriately. The oral health practitioners at the MDU were observed speaking affectionately about long-term patients, and remembering everyone’s names. Others were observed tailoring their advice to the needs of the service users, and letting service users tell their stories about their lives, occasionally attempting to bring the conversation back to oral health. In some instances, the oral health practitioner would share their own life experiences if it was related to the topics that were being discussed:

*“When a service user said he found it difficult to stop smoking, Practitioner 1 admitted that she was an ex-smoker, and explained that she still feels tempted”*

*(Observation 1)*

The oral health practitioner in Board 3 demonstrated on numerous occasions that she was willing to go the extra mile for the service users she sees. These extra tasks that she does are not because she has been asked to by her managers, but because she cares about the service users, and wants to offer them the best service she can.

*“To me it seems that Practitioner 4 goes above and beyond for the service users she sees. She will offer to phone and make appointments, sends them reminders the day before (even on her day off) and she will even take them to an appointment. It’s clear that she cares if they attend or not—she mentioned that she has asked some practices to waive fines, and clearly advocates for the service users when necessary.”*

*(Observation 8)*

Incentives were also used, to encourage service users to engage with the oral health practitioners, aiding in the development and maintenance of the working alliance. These were predominantly toothbrush and toothpaste packs, but in some NHS Boards could also be free samples of a wider range of toothpastes (e.g., Oral-B or Corsodyl), denture-care packs, or toothbrush cases. Indeed, service users were always eager to get the free samples of the branded toothpastes, compared to the more basic NHS-provided packs. These incentives often acted as an icebreaker, particularly in locations where the oral health practitioner had to approach service users to see if they were interested in discussing oral health, rather than interested service users approaching them. In some instances, when a wide range of resources were available, they were also an opportunity for the oral health practitioners to find out more about the service users oral health—they could ask questions under the pretence of ensuring they gave them the most appropriate product. For example:

*“Practitioner 1 would take the time to find free samples that would be specific to the service user, e.g., Corsodyl toothpaste for people with bleeding gums, or denture care items”*

*(Observation 1)*

### 3.2. Alliance 2: Oral Health Practitioners and Third Sector Staff

While considering Alliance 1, it became apparent that Smile4life delivery may be reduced if there is no alliance between the oral health practitioners and the Third Sector staff. The cooperation of the Third Sector staff is crucial as they allow the oral health practitioner access to their service and their service users. For example, they can choose to promote Smile4life and visits from the oral health practitioner or do nothing to encourage service users to be interested in their oral health. In the observations, the Third Sector staff often acted as a barrier to alliances forming between them and the oral health practitioners, as well as between the oral health practitioners and the service users. Alternatively, in one NHS Board, the oral health practitioners themselves were a barrier to a working alliance with local Third Sector staff. These barriers are discussed in more depth below.

#### 3.2.1. Barriers

In Board 1, there was no observed working alliance between the oral health practitioners, service users or Third Sector staff. Unlike the more pro-active oral health practitioners observed in the other Boards, in Board 1, the oral health practitioners did not approach service users—they offered a mobile dental unit and if any service user was interested in getting treatment they had to approach the unit.

*“Practitioner 3 did not really interact with service users beyond telling them if they can be seen by the dentist, or making general conversation. She would encourage people to wait in the drop-in rather than in the MDU before their treatment”*

*(Observation 3)*

*“The oral health team do not seem bothered to recruit any patients, even if that means sitting waiting with nothing to do—the feeling seems to be that if a patient wants to be seen then they will come to the MDU.”*

*(Observation 4)*

In the other two NHS Boards, in most instances, the oral health practitioner was reliant on the Third Sector staff at the organisations to promote and advertise the oral health visits, e.g., by putting up posters, flyers, or announcing the visit over the overhead speaker. However, this was often not the case:

*“In all three establishments today, staff did not seem well prepared for Practitioner 2’s visit—her poster was only displayed in one of them, and they had not spoken to their service users about her visit…In one place, the room we were offered was in the staff area, so there would never be any passing service users”*

*(Observation 2)*

This excerpt from the field notes highlights the extent to which the oral health practitioner is at the mercy of the Third Sector staff—during that observation, the oral health practitioner visited three services and only spoke to one service user. At the first service, the oral health practitioner had been advised to attend at a time when there were no service users awake; at the second, the oral health practitioner was given a room to use which was a meeting room in the staff area. There was also no attempt by Third Sector staff at any of the three locations to let their service users know that Practitioner 2 was available to talk to. This suggests that, if the oral health practitioner does not know the staff that well, they are not motivated to prioritise her requests or to put up her posters. Similarly, if they do not understand the importance of oral health then they would be unlikely to encourage their service users to care about their oral health.

#### 3.2.2. Enablers

Despite the barriers discussed above, any potential alliance between the oral health practitioner and the Third Sector staff was dependent on both parties engaging with each other. While the previous example demonstrated that, when the Third Sector staff do not help the oral health practitioners, Smile4life cannot be delivered and it is the responsibility of the oral health practitioner to attempt to establish a relationship in the first instance. As with forming an alliance with service users, the oral health practitioners were themselves an enabler to forming relationships with the Third Sector staff.

*“it is clear that Practitioner 1 works hard at maintaining strong relationships with staff at these locations… she makes a point of visiting every 6 weeks and reminds them the day before that she will be visiting.”*

*(Observation 1)*

*“Practitioner 1 clearly has a good relationship with the staff… she told me that ‘keeping the staff sweet’ is a major part of her role and really helps with building rapport”*

*(Observation 7)*

As with service users, incentives also aided in the formation of an alliance with the Third Sector staff—Practitioner 1 said as much when she told the researcher that she needed to “keep the staff sweet” so would purposively keep two packs of PolyGrip aside for one member of staff in particular (Observation 7).

### 3.3. Alliance 3: Third Sector Staff and Service Users

The third alliance that exists between the three key parties involved in Smile4life is the one between the Third Sector staff and the service users. However, interactions between the Third Sector staff and the service users were not observed during the present study as the oral health practitioners were the focus of the participant observation—this, therefore, represents a limitation with regard to dental care policy. The need remains to conduct additional studies to confirm this process to provide dental care for people experiencing homelessness.

## 4. Discussion

The findings from the observation sessions suggest that key factors in the delivery of Smile4life are the working alliances among the oral health practitioners, the Third Sector staff and the service users.

Within this triad, there are three key alliances: (1) oral health practitioners and service users; (2) oral health practitioners and Third Sector staff; and (3) Third Sector staff and service users. When there are strong working alliances, the Third Sector staff can promote and signpost to the oral health practitioner, who in turn can engage directly with service users about their oral health.

With regard to Simmel’s work concerning coalitions within a triad, in an ideal Smile4life scenario, the Third Sector staff would act as a “non-partisan”, facilitating a connection between the oral health practitioner and the service users [30]. Indeed, this was the case in the more successful interactions that were observed, However, it is apparent from the observation data that this was not always the case: while not necessarily partisan, it would appear that some Third Sector staff were indifferent about oral health. In these instances, it is possible that the Third Sector staff interpreted the oral health practitioners as intruders, disturbing their existing dyadic relationship with their service users, or potentially excluding them from the triad. Due to this possible interpretation, in many cases, it is the Third Sector staff that hold the power within the triad—they can control access to the service and the service users, essentially acting as gatekeepers to protect the service users from what they may perceive as a threat, i.e., the “tertius gaudens” scenario as described by Simmel [30].

However, in instances where the Third Sector staff are helpful—and perhaps the communication between parties is more effective—there can still be the issue of disinterested or disruptive service users. In these cases, the power distribution shifts and it is the service users that hold the power—it is up to them if they are receptive to Smile4life, or whether they will be rude and disinterested in what the oral health practitioner has to say. For example, in instances such as those observed in Boards 1 and 2, the service users demonstrated their power by being disruptive or argumentative with the oral health practitioners, e.g., arguing about fluoride.

Interestingly, in both scenarios, the oral health practitioners are powerless. Indeed, in many respects, they *are* intruders, or outsiders, attempting to infiltrate the service where the Third Sector staff and the service users are based. In this respect, when the oral health practitioner enters, they allow a majority to form, i.e., two against one [29]. Caplow examined the power dynamics and coalitions present in three-person groups and considered there to be eight types of coalition, dependent on the power held by each of the three parties, e.g., A = B = C, where all parties are equal; or A < B, B = C, where B and C have equal power, which is greater than that of A—this could be said to be the case when oral health practitioners are seen as intruding upon the existing alliance of the Third Sector staff and the service users [31]. 

However, due to the scope of the research and the focus on the oral health practitioners, it was not possible to observe Alliance 3, and therefore not possible to fully explore the different power dynamics present in the three-party group. This is a limitation of this research, and a potential topic for future studies in this area. Observing the relationship between the Third Sector staff and the service users would reveal if oral health is considered a priority for these individuals, particularly in the absence of the oral health practitioners. It would also aid in a deeper understanding of how the triadic working alliance operates, and if the entrance of the oral health practitioners is truly seen as an intrusion, forcing the three-person group into “a pair and an other”, with the oral health practitioner being the “other” [32] (p. 351) [30].

There are also limitations associated with participant observation as a data collection tool. While it allows the researcher to gain first-hand experience of the topic being studied, it is not repeatable, and is dependent on the researcher’s interpretations of what is being observed [33]. It is also time-consuming, requiring the researcher to spend long periods with the participants being observed [22]. However, despite these limitations, it was an appropriate tool for this study, as it allowed the authors to immerse themselves in the working lives of the oral health practitioners, which would not necessarily have been possible with other data collection methods, e.g., interviews. While it was time-consuming, this provided the “time to develop an intuitive feel for the particular system studied” [33] (p. 37).

While the observations concerned the delivery of the Smile4life intervention and the relationships between oral health practitioners, homeless service users and Third Sector staff, it is possible that the results could be generalisable to any situation where oral health is being delivered to an excluded or vulnerable population, such as adults with learning disabilities. Many of the barriers faced by the oral health practitioners could be considered organisational barriers, which are not specific to the homelessness context (e.g., uncooperative Third Sector staff and lack of access to service users/patients). Similarly, the factors identified as enablers to the alliances discussed above (e.g., the skills and attitude of the oral health practitioners) would be beneficial to any oral health practitioner attempting to forge a good working relationship with any patients or organisations, not just within homelessness.

With regard to recommendations for the continued delivery of the Smile4life intervention, based on the observations, it would appear that there needs to be a strengthened relationship—or alliance—between the oral health practitioners and the Third Sector staff. Specifically, there should be more awareness raising about the benefits of Smile4life and what the oral health practitioners are attempting to do when they visit a Third Sector organisation. This would potentially overcome the issue of the location or physical space the oral health practitioner is given to deliver Smile4life. Additional buy-in from the Third Sector could also facilitate improved access to the service users, if the Third Sector staff see the importance of oral health and encourage their service users to see the oral health practitioner. However, it must be acknowledged that some barriers experienced by the oral health practitioners are organisational issues, such as staffing within the Third Sector organisation, and as such are not straightforward to overcome. In addition, it would be necessary to investigate the barriers and enablers in more detail, to establish what the oral health practitioners themselves think of their experiences and their role in delivering Smile4life, before any changes are recommended regarding the delivery of Smile4life.

## 5. Conclusions

The observation sessions have demonstrated how Smile4life is implemented in three different NHS Boards across Scotland, and highlighted the variations in practitioners’ approaches to their Smile4life-related work. Furthermore, they revealed the three key working alliances that exist among the oral health practitioners, the Third Sector staff and the service users. By referring to theories of triadic coalitions, it was possible to infer the types of relationships that exist within the triad, and the power dynamics that exist within these relationships. To successfully deliver Smile4life to service users, all parties in the triad must work together, and each of the three key alliances must be strong. There can be no Alliance 1 if Alliances 2 and 3 do not already exist. In addition, there were many factors that influenced these alliances, and these acted as barriers and enablers to strong and beneficial relationships.

## Figures and Tables

**Table 1:** Details of observation sessions.

Observation	Location	Services Visited	Setting
Observation 1	Board 2	3 supported accommodation establishments (1 h each)	Shared living room Meeting room
Observation 2	Board 2	An accommodation for young men (1 h) A long-term accommodation for families and individuals (1 h) A short-term accommodation for young people (1 h)	Kitchen Meeting room Shared living room
Observation 3	Board 1	Mobile dental unit at a drop-in service (2 h)	Mobile dental unit waiting area
Observation 4	Board 1	Mobile dental unit at a harm reduction service for young people (1 h 30 min)	Mobile dental unit waiting area
Observation 5	Board 1	Mobile dental unit at a harm reduction service for young people (1 h 30 min)	Mobile dental unit waiting area
Observation 6	Board 2	Drop-in service providing hot meals (3 h)	Canteen
Observation 7	Board 2	A space for families and friend of prisoners to wait before entering the prison (4 h 30 min)	Visitor centre
Observation 8	Board 3	An emergency accommodation for women (1 h) A support and drop-in service (1 h 45 min) An emergency accommodation for men (2 h)	Canteen Reception area
Observation 9	Board 3	A homeless assessment centre and short-term accommodation (2 h) Supported long-term accommodation for women (1 h)	Medical room Service user’s flat

**Table 2:** Details of participating oral health practitioners.

Practitioner Number	Board	Job Title	Gender
Practitioner 3	Board 1	Dental Health Support Worker	Female
Practitioner 1	Board 2	Oral Health Educator	Female
Practitioner 2	Board 2	Oral Health Educator	Female
Practitioner 4	Board 3	Dental Health Support Worker	Female
